# Folie a Deux in 2 Elderly Sisters - Challenges in Diagnosis and Management

**DOI:** 10.1192/j.eurpsy.2023.2260

**Published:** 2023-07-19

**Authors:** K. H. B. Suhairi

**Affiliations:** 1 Insitute of Mental Health Singapore, Singapore

## Abstract

**Introduction:**

Folie a deux is a rare psychiatric disorder where an identical or similar mental disorder affects two or more individuals usually with close emotional ties. Management of patients with folie a deux may require multiple types of treatment including separation, family therapy and antipsychotics.

**Objectives:**

Narrative case of Folie a deux in 2 elderly sisters

**Methods:**

Clinical case presentation through retrospective review of clinical notes

**Results:**

We present a case of 2 sisters living together of 76 and 74 years of age. The elder sister, Sister A, presented to Singapore’s Institute of Mental Health on October 2021 after she was referred from court for disturbed behaviour with more than 10 years history of paranoid delusions against their neighbours, eventually extending to strangers in general. She was admitted and stayed in hospital from October 2021 to February 2022. She was referred to community psychiatric services to perform home visits due to her unwillingness to attend subsequent follow ups after discharge. During these home visits, it was determined that her sister, Sister B, was displaying delusional thinking as well. Sister A was started on antipsychotics while Sister B was managed non-pharmacologically. After commencement of antipsychotics, Sister A showed good response with improvement of symptoms, while Sister B continued to display delusional thinking. Discussion was made with the next-of-kin of both sisters, their nephew, and the decision was made to continue to observe these symptoms and monitor for their resolution with continued treatment of Sister A. A multidisciplinary approach was also used to attempt to reintegrate them into society to rebuild trust in the public. Currently both sisters still report vague paranoia of strangers in general and a multidisciplinary team is involved in improving their symptoms further.

**Conclusions:**

With this case report, we hope to raise awareness of the different methods that are useful in the management of Folie a deux along with the challenges faced when managing these cases.

**Disclosure of Interest:**

None Declared

